# Horseshoeing1Read before the Missouri Valley Veterinary Medical Association.

**Published:** 1900-06

**Authors:** Charles Saunders

**Affiliations:** El Dorado, Kan.


### HORSESHOEING.[1]

By Charles Saunders, V.S.,
EL DORADO, KAN.

The subject of practical horseshoeing embraces a wider field for combined labor than may be apparent under its title. To approach proficiency a clear understanding of the locomotive functions of the horse is required, and if that be acknowledged the necessary steps must be taken by going back to the elements of anatomical science.

The practice of farriery, in which, as a whole, science, artistic skill, and physical activity are called into requisition, demands resources of self-denying, able men.

Man, unlike every living creature besides, works to-day by the light of past ages, and owes to his fellows and to future generations the obligation to use his talents to the utmost in forwarding the cause of truth in everything he undertakes; whilst the lower animals, having no such mission, act by instinct, the bee of to-day following the same laws as those of his species in all preceding generations.

[1] Read before the Missouri Valley Veterinary Medical Association.

Few persons appreciate the importance of horseshoeing, while a small number tell us it is unnecessary. The value of horseshoeing depends upon the manner in which it is done. Very seldom does the owner of horses appreciate the quality of the work. As a rule, the price charged or the distance from forge to the stable regulate the choice of the farrier. Such matters should not be allowed to decide between one farrier and another. A bad workman may do an injury at one shoeing which would cost the owner of the horse more than would pay ten times over the difference between his charges and the higher price of a better man.

Shoeing is by no means the simple affair it appears to the non-professional mind. If there is one thing more than another which has a tendency to technology, it is the art of horseshoeing, for a shoer to have a knowledge of the structure and functions of the limb and foot, the preparation of the foot, the various kinds of shoes, to make fit and attach them properly, and then give a reason for his work is, I think, one of the finest examples of technology or "science with practice."

An art, therefore, which has so much influence for good or evil, so far as usefulness and comfort of the horse are concerned, surely deserves the serious study of all those who are interested in that noble animal. A good system, founded on the teachings of anatomy and physiology, and perfected by daily experience, must prove of immense benefit to horse and owner, while a bad system, conducted in ignorance or carelessness, cannot but bring about pain and speedy uselessness to the animal and loss to the owner.

The old proverb, that for the want of a nail the shoe was lost, and for the want of a shoe the horse was lost, and for the want of a horse the man was lost, has been illustrated times without number. Few persons, however, are aware of the terrible consequences which have more than once attended neglect in the shoeing of horses.

Napoleon's retreat from Moscow depended for most of its hardships and horrors on the want of proper shoeing. The horses could not keep their feet, and were unable to drag the guns and wagons, which had to be abandoned. During the Franco-Prussian war Bourbaki's retreat became a confused rout from a similar cause. In the Civil War we experienced many hardships and loss for the want of horseshoers and material. In the Philippines, the past season, our troops (one company of them) had to abandon their horses for the want of horseshoers.

History, though much debated, it is still uncertain to whom or even to what race were the first to shoe horses with nailed-on shoes. The exact time when shoes were applied to the horses' feet is not known. The Celts, however, are credited, especially by French investigators, with having employed nailed-on shoes before the opening of the Christian era, and having extended their use throughout Gaul, Germany, and England. The Gauls were the first to practice the art of horseshoeing. About the fifth century in Rome we find horseshoes which began to approximate to those used to-day.

William the Conqueror is said to have introduced the art of shoeing into England. He gave to Simon St. Liz, a Norman, the town of Northampton and the hundred of Falkley, valued at $176 per annum, to provide shoes for his horses; and Henry de Firrers, who also came over with him, he appointed superintendent of the shoers, whose descendants, the Earls of Firrers, have six black horseshoes on a silver ground in the quartering of their arms. Their Castellan, at Oakham, in Rutlandshire, the seat of this family, a singular and rather tyrannical custom long prevailed, has the privilege of demanding one of his horseshoes, unless he chose to redeem it by a fine, from every nobleman or baron of the kingdom on his first journey through the town. The shoes, together with the giver's name, are affixed to the door of the castle. This right is still practised.

The farriers of London, England, celebrate the 23d of November by drinking pearl, a mixture of ale, eggs, and spirits made hot, in memory of Old Clements, who was the first man to make a shoe and nail it on, and for all that I know the first horseshoer to strike not for wages, but for honor. During the building of the Tower of Babel the horses and mules became sore-footed, so they abandoned them. Necessity being the mother of invention, Old Clements invented horseshoeing, shod the horses and mules. It pleased the king (Nimrod) so much that he gave a grand banquet to his nobles. This displeased Old Clements, and he felt insulted and struck; the horses and mules became lame for the want of shoeing; work on the Tower was suspended to a certain extent. The king wanted to know the cause of this; was informed that Old Clements felt insulted and had struck. The king then gave a grander banquet than before in honor of Old Clements; had him sit on the right-hand side of him, and presented him with a white sheepskin apron, with gold tassels on the two lower corners, and that is the way we distinguish a farrier from a blacksmith when we meet him away from the forge with his apron on; only the corners are fringed instead of gold tassels.

The Patent Office has about two hundred horseshoes, and not one of them is of any practical use unless it be nailed over a barn door to keep the poor witches from entering, and good luck for the horse that it was used that way instead of torturing him by applying it to his foot. The horseshoers are not behind other trades in discoveries and inventions.

The ancients first used Spanish broom-corn plaited over the bottom of the foot, the ends carried up the leg, bound on with cords of raw-hide; next leather was used the same way, then leather with brass, or a kind of brass was fastened on the soles, made fast to the hoof by straps; next they fastened plates of metal to the hoof by straps. From that on they fastened the shoes on by nails. All kinds of material have been used for shoes. Gold, silver, bronze, brass, aluminum, cork, wood, straw, paper, rubber, gutta-percha, felt, cloth, horn, oakum, iron, and steel have been used either alone or in combination.

A study of the specimens surprises and somewhat lessens the self-esteem we may have indulged in at our progressiveness, for after all many of our supposed new ideas are only resurrections.

